# Poly(Lactic Acid) Blends with Poly(Trimethylene Carbonate) as Biodegradable Medical Adhesive Material

**DOI:** 10.3390/ijms18102041

**Published:** 2017-09-28

**Authors:** Shuang Zhang, Hongli Li, Mingwei Yuan, Minglong Yuan, Haiyun Chen

**Affiliations:** Engineering Research Center of Biopolymer Functional Materials of Yunnan, Yunnan Minzu University, Kunming 650500, China; zhshu@deakin.edu.au (S.Z.); honglili_1982@163.com (H.L.); yuanmingwei@163.com (M.Y.)

**Keywords:** Poly(Lactic Acid)-Poly(Trimethylene Carbonate), medical adhesive, avoid infection, hemostasis

## Abstract

A novel medical adhesive was prepared by blending poly(lactic acid) (PLA) with poly(trimethylene carbonate) (PTMC) in ethyl acetate, and the two materials were proven to be biodegradable and biocompatible. The medical adhesive was characterized by ^1^H nuclear magnetic resonance (^1^HNMR), gel permeation chromatography (GPC), scanning electron microscopy (SEM) and differential scanning calorimetry (DSC). The water vapor transmission rate (WVTR) of this material was measured to be 7.13 g·cm^−2^·24 h^−1^. Its degree of comfortability was confirmed by the extensibility (E) and the permanent set (PS), which were approximately 7.83 N·cm^−2^ and 18.83%, respectively. In vivo tests regarding rabbit immunoglobulin M (IgM), rabbit immunoglobulin G (IgG), rabbit bone alkaline phosphatase (BALP), rabbit interleukin 6 (IL-6), rabbit interleukin 10 (IL-10), rabbit tumor necrosis factor α(TNFα), glutamic-oxaloacetic transaminase (AST/GOT), glutamic-pyruvic transaminase (ALT/GPT), alkaline phosphatase (AKP), blood urea nitrogen (BUN) and creatinine (Cr) indicated that the PLA-PTMC medical adhesive was not harmful to the liver and kidneys. Finally, pathological sections indicated that PLA-PTMC was more effective than the control group. These data suggest that in addition to having a positive effect on hemostasis and no sensibility to wounds, PLA-PTMC can efficiently prevent infections and has great potential as a medical adhesive.

## 1. Introduction

Each year, millions of people suffer from many types of wounds, including traumatic or surgical wounds, which require a proper closure. To develop better materials for the treatment of wounds, the translation of modern biomaterials from the lab to the clinical hospital will permit an in-depth understanding of how biomaterials interact with biological systems at both cellular and molecular levels, with the ultimate purpose of creating more suitable materials and products [1,2,3,4,5,6,7].

Previously, there have been many techniques for curing a wide range of wounds, such as sutures and staples. However, although sutures can provide great tensile strength and show relatively low failure rates [8,9], the disadvantages of suturing are that it is time consuming, is not always technically possible, thus requiring anesthesia, and induces undesirable scar formation [9,10]. Staples easily damage surrounding tissues while evoking an inflammatory response and causing scar tissue formation. Most importantly, the use of staples also results in a significant failure rate [8,9,11]. Robust adhesion and cohesive integrity are valuable characteristics of a medical adhesive, especially for applications requiring a long-term performance of the material. However, the limitations of currently-approved synthetic adhesives include poor adhesion in the presence of biological fluids, sensitization, an allergic response and inflammation. They can always cause problematic infections, which can cause many types of diseases. Thus, development of new biomaterials with a low cost, low toxicity and less infection is quite essential.

Poly(lactic acid) (PLA), apart from being derived from renewable resources (e.g., corn, whey [12,13], wheat and rice), is biodegradable, recyclable and compostable [14,15]. Its production also consumes carbon dioxide [16]. Due to its ability to be degraded and assimilated inside the human body within a few months, its first applications were in the biomedical field [17,18,19,20]. Moreover, PLA degradation products are non-toxic at a lower composition, making it a natural choice for biomedical applications [21,22]. Furthermore, PLA requires 25–55% less energy to produce than do petroleum-based polymers, and estimations indicate that this can be reduced further to less than 10% in the future [23]. Although it is an ideal biomaterial with biocompatibility and biodegradability, PLA is a very brittle material with less than 10% elongation at break [24,25], which largely limits its practical use in the medical field.

Blending of PLA with other high strength materials is one of the most extensively-used methods to improve the mechanical properties of the material. To overcome the disadvantages mentioned above, PLA has been blended with some biodegradable polymers such as poly(para-dioxanone) [26], poly(propylene carbonate) [27], poly(butylene succinate) and derivatives [28,29] to improve its mechanical properties, especially toughness. Among these, PTMC is an amorphous polymer with a glass transition at 12 °C with low crystallinity and high mechanical properties [30], and it has good biocompatibility and biodegradability [31,32]. High-molecular-weight PTMC maintains elastic properties at ambient temperature [33,34,35], and the mechanical performance of PLA can be improved efficiently.

Therefore, the aim of this study was to produce and characterize PLA-based, high-performance biocompatible composite materials for biomaterial applications such as medical adhesives. This novel synthesized composite contains different percentages of PTMC to enhance the properties of the polymer. Medical adhesives with different ratios of PLA and PTMC were first produced and characterized by ^1^H nuclear magnetic resonance (^1^HNMR), gel permeation chromatography (GPC), scanning electron microscopy (SEM) and differential scanning calorimetry (DSC). The film-forming time, water vapor transmission rate, degree of comfortability and water contact angle were measured afterwards.

A ratio of 7:3 of PLA:PTMC was found to have the most suitable properties, and animal experiments were conducted to ensure the performance of this product. In the experiment, 10 rabbits with similar weights were separated into two groups: the PLA-PTMC group and the positive control group. Then, a wound model was created and observed, and the wounds’ status was recorded until the wounds recovered. During this period, glutamic-oxaloacetic transaminase (AST/GOT), glutamic-pyruvic transaminase (ALT/GPT), alkaline phosphatase (AKP), blood urea nitrogen (BUN) and creatinine (Cr) were measured to determine the influence of the medical adhesives on hepatic and renal tissues, and enzyme-linked immunosorbent assay (ELISA) assays were performed to determine the content of immunoglobulin M (IgM), rabbit immunoglobulin G (IgG), rabbit bone alkaline phosphatase (BALP), rabbit interleukin 6 (IL-6), rabbit interleukin 10 (IL-10) and rabbit tumor necrosis factor α(TNFα). The results suggests that this type of biomaterial can efficiently reduce infections because it can form a thin and ventilated film quickly on the surface of the skin and promote wound repair, with less harm to other organs.

## 2. Results and Discussion

### 2.1. Characterization of Medical Adhesive Films

**Figure 1:**
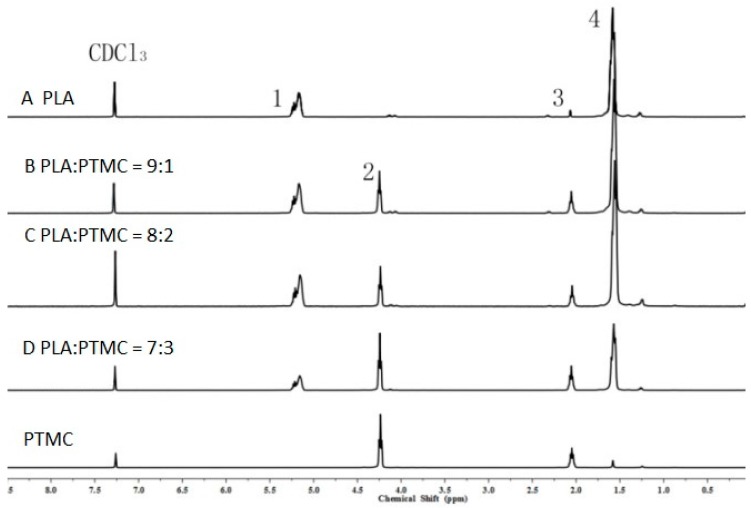
^1^H nuclear magnetic resonance (^1^HNMR) spectra of poly(lactic acid) (PLA) and poly(trimethylene carbonate) (PTMC) and their blends.

**Figure 2:**
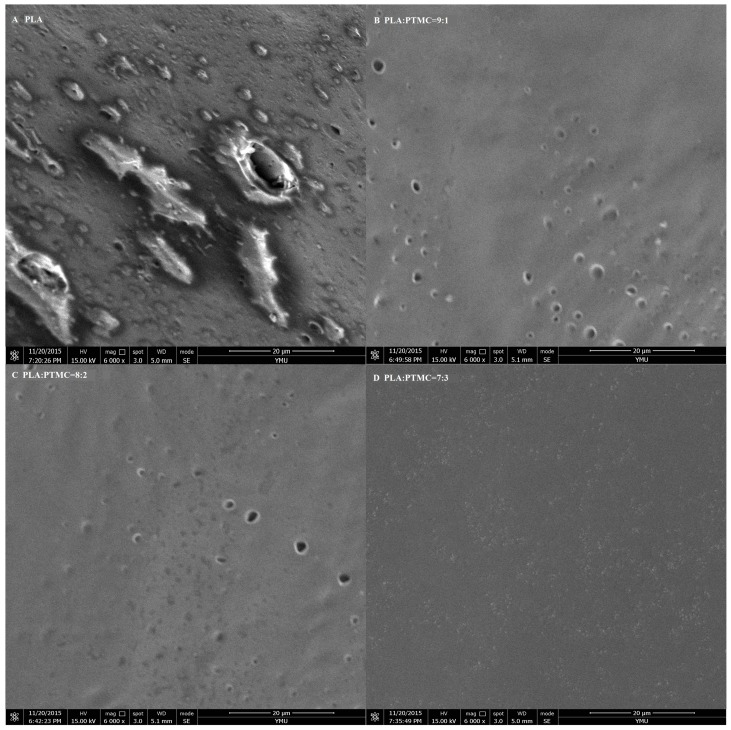
SEM micrographs of medical adhesive with different ratios of PLA-PTMC. (**A**) PLA; (**B**) PLA:PTMC = 9:1; (**C**) PLA:PTMC = 8:2; (**D**) PLA:PTMC = 7:3. Scale bar: 20 µm.

**Figure 3:**
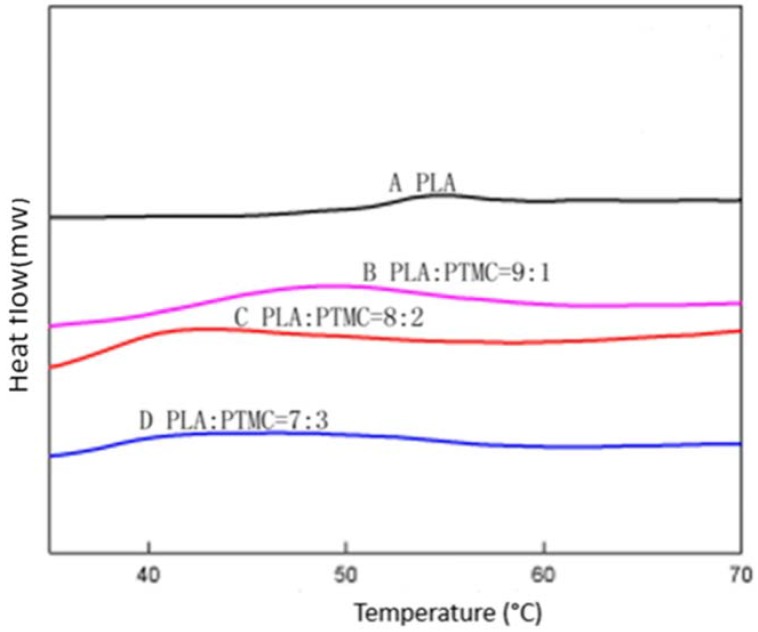
Differential scanning calorimetry (DSC) of different ratios of PLA-PTMC.

To characterize the properties of the medical adhesive and choose the most suitable ratio of PLA/PTMC, poly(lactic acid) (PLA, *M*w = 280 kDa, *M*w/*M*n = 1.98) was modified with poly(trimethylene carbonate) (PTMC, *M*w = 100 kDa, *M*w/*M*n = 1.70) in different ratios of 9:1, 8:2 and 7:3 to facilitate their use in biomaterial studies. A solvent evaporation technique with 50 mL of chloroform was used to prepare thin films to be characterized by ^1^HNMR, GPC and SEM (from Figure 1, Figure 2 and Figure 3 and Table 1). From the GPC data, when the content of PTMC increased, the polydispersity index (PDI) of the blends decreased (see Table 1). The raw polymer and blends exhibited high molar masses ranging from 98,972–77,253, and the PDI of the blends ranged from 2.05–1.85. The chemical compositions of the blends were determined by ^1^HNMR analysis. As shown in Figure 1, Peak 1 and Peak 4 are assigned to lactyl CH and CH_3_ groups, respectively, and Peak 2 and Peak 3 are assigned to the trimethylene carbonate (TMC) CH_2_ group. The different ratios of PLA and PTMC are presented with the different peak intensities. SEM was used to track changes in the film surface morphology among different blends. Figure 2 shows the SEM photographs of A, B, C and D; with an increased scale of PLA-PTMC, the composite looked smoother than pure PLA; namely, the blends became better distributed when the ratio of PLA:PTMC was 7:3 [36]. Figure 3 shows the DSC thermograms of PLA and PLA-PTMC blends. The glass transition (*T*g) of the PLA was observed at approximately 58 °C; the same result was achieved in Martin’s work [37]. This is consistent with PLA having a *T*g between 50 and 80 °C [38,39,40]. With increasing PTMC content of the polyurethane, the *T*g of the polymers decreased. When the ratio of PLA:PTMC was 7:3, the *T*g was measured as 38.8 °C, which is lower compared with pure PLA, due to the distribution of PTMC in the film. Because the glass transition of PTMC is around −20 °C [32,41,42], the addition of PTMC made the glass transition of PLA decrease.

### 2.2. Evaluation of Physical Properties

**Figure 4:**
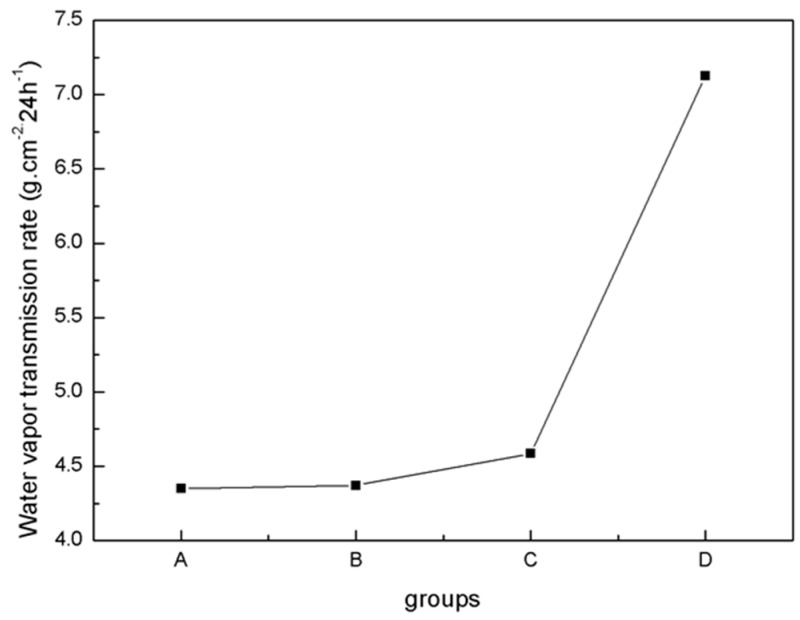
Water vapor transmission rate determined using the cup method.

**Figure 5:**
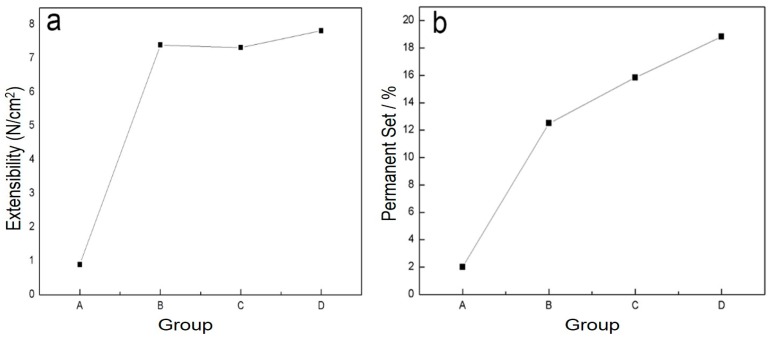
The comfortability of the blends was assigned through extensibility (**a**) and permanent set (**b**).

The physical properties of blends of different ratios of PLA/PTMC were evaluated using film formation time, water vapor transmission rate, the comfortability degree and the contact angle. With the addition of PTMC, the film formation time increased sharply; however, when the ratio of PLA-TMC decreased from 8:2–7:3, the film formation time increased slightly, from 60.33–62.33 min. Figure 4 shows that the water vapor transmission rate of the sample rose sharply, from 4.59–7.13 g·cm^−2^·24 h^−1^ with a ratio change from 8:2–7:3. Additionally, the water vapor permeability of the films is better than the PLA film reported in the literature [36,43]. The result that air permeability increased may be caused by the increasing content of PTMC in the composite. Additionally, based on the extensibility (E) and permanent set (PS), the comfortability of the material was evaluated. Just as Figure 5 presented, with the increasing content of PTMC in the composite, the comfortability of the medical adhesive was improved. Additionally, when the ratio of PLA:PTMC went up to 7:3, the comfortability of the material rose to the highest, and the extensibility and permanent set reached 7.83 N·cm^−2^ and 18.83%, respectively. This indicates that a ratio of PLA: PTMC of 7:3 was the optimal choice for a medical adhesive. The contact angle indicates that when the ratio of PLA-PTMC reached 7:3, the hydrophobicity was the highest, which is desirable for preventing wound infections. This is presented in Table 2.

### 2.3. Biological Performance

**Figure 6:**
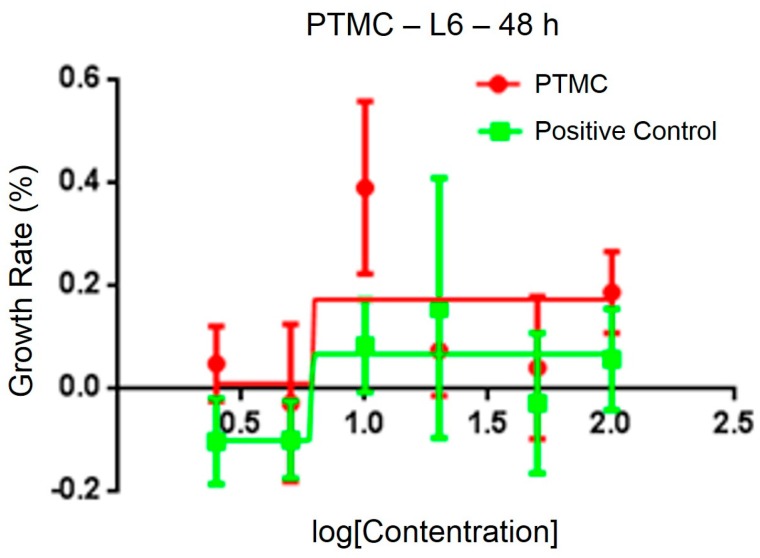
Cytotoxicity of the medical adhesive determined by 3-(4,5-dimethylthiazol-2-yl)-2,5-diphenyl tetrazolium bromide (MTT).

After testing the physical and chemical properties, the optimal sample was chosen for animal experiments. The sterility test of PLA/PTMC at a ratio of 7:3 demonstrated no obvious colony formations after 36 h, and the cytotoxicity of the medical adhesive was evaluated using 3-(4,5-dimethylthiazol-2-yl)-2,5-diphenyl tetrazolium bromide (MTT). The IC_50_ of a PLA/PTMC medical adhesive with a ratio of 7:3 was determined to be 6.24 μg/mL, according to linear regression analysis of the information provided in Figure 6. In some research, the IC_50_ of some polymers was smaller than 10 µg/mL [44,45]. When comparing with the positive group, the IC_50_ value was smaller. That is to say, the growth rate was higher in the experiment group than the positive group as presented in Figure 6. Kulkarni reported that PLA was non-toxic, and Schakenraad presented that PLA was tissue compatible [23,46]. Furthermore, after the ears were covered with the medical adhesive for 2, 4, 8, 12 and 24 h, the ears of the rabbits did not display any aberrant phenomenon. These preliminary results suggest that this type of medical adhesive can be used as a biomaterial.

### 2.4. The Result of Animal Experiments

**Figure 7:**
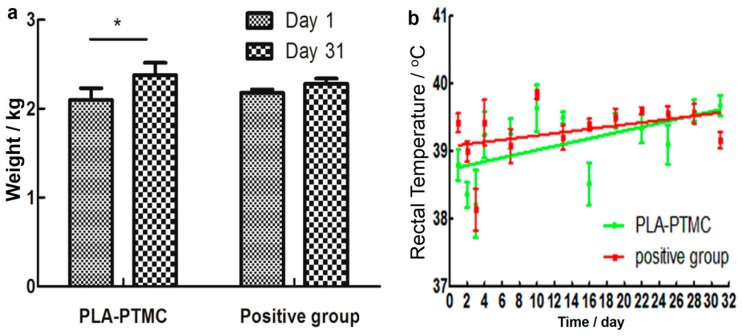
Weight and temperature changes of the rabbits during the experiments. (**a**) Presents the change of weight during the experiment; (**b**) shows the rectal temperature of the rabbits. “*” present there exists statistic difference.

The result of the skin irritability tests suggests that PLA-PTMC as a medical adhesive does not trigger allergies. According to the data (from the first day of establishing the wound model to the last day of wound recovery), the weight of the rabbits showed an increasing trend during the experiments, as shown in Figure 7a, particularly in the PLA-PTMC group, with a 0.16 kg average. Figure 7b shows that one fever occurred during the experimental process with PLA-PTMC and the positive control group. However, this phenomenon lasted only one day.

**Figure 8:**
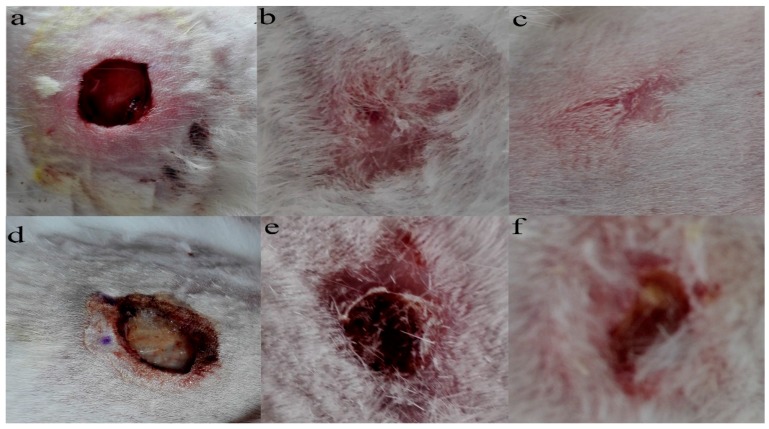
The records of the wound during different periods after being covered by PLA-PTMC and the positive group medical adhesive. (**a**–**c**) represent the state of the wound covered by PLA-PTMC medical adhesive after 1, 20 and 31 days, and (**d**–**f**) show the situation of the positive group at the same time, respectively.

**Figure 9:**
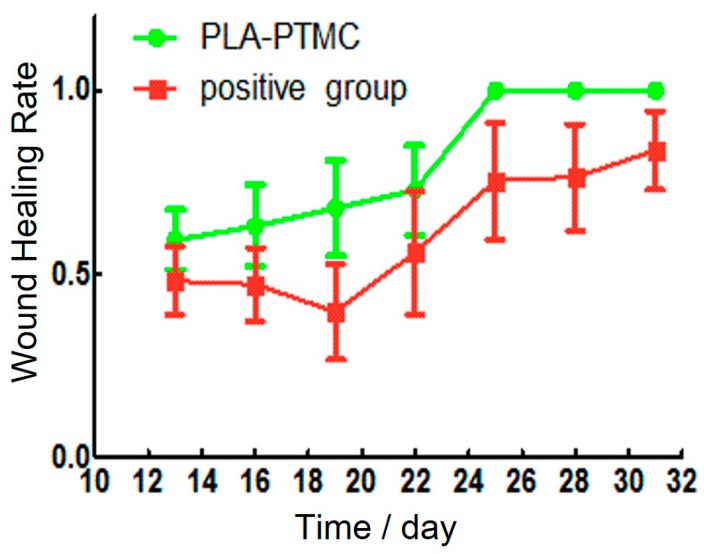
The wound healing rate of the PLA-PTMC group and the positive control group.

Granulation tissue was observed to grow on the sixteenth day after the operation. The wounds in the PLA-PTMC group were completely healed on the 30th day, but the positive control group had residual wounds of 0.4 cm × 0.3 cm that had not healed fully. Figure 8a–c are images of the wounds that were covered by PLA-PTMC after 1, 20 and 31 days after the operation, respectively, and Figure 8d–f are images of the positive group. Figure 9 shows the wound healing rate from the 13th–31st day with PLA-PTMC and the positive group. The wound healing rate for the PLA-PTMC group was significantly higher than that of the positive group (*n* = 5).

The influence on hepatic functions of rabbits in the PLA-PTMC and the positive control groups was evaluated based on the serum levels of ALT/GP, AST/GOT, AKP, BNU and creatinine, which were determined using the appropriate kits. The statistics suggested that AKP activity and BNU concentrations were significantly different for the PLA-PTMC medical adhesive, whereas the positive medical adhesive and other indicators produced no obvious significant difference.

**Figure 10:**
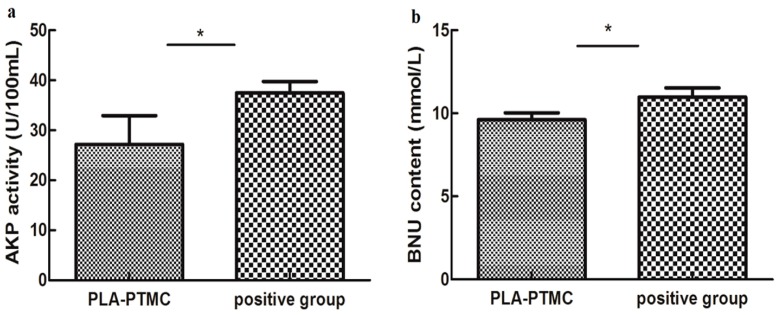
The AKP (alkaline phosphatase) activity (**a**) and the blood urea nitrogen (BUN) content (**b**) of PLA-PTMC and the positive group. Values are the means ± S.E.M. (*n* = 5). * *p* < 0.05 compared with the control group; * *p* < 0.01 compared with the control group; * *p* > 0.05 was considered not to be statistically different. These indicators were detected using different kits that were obtained from the Nanjing Jiancheng biological technology limited company (Nanjing, China).

Figure 10a shows the AKP activity using PLA-PTMC and a positive medical adhesive, and Figure 10b presents the BUN content. BUN is used as a surrogate marker of neurohormonal activation, and AKP has been employed as one indicator in the very sensitive enzyme-linked immunosorbent assay (ELISA) [46]; it is one of the most commonly-assayed enzymes in clinical practice to diagnose different types of diseases [47]. Figure 10a shows that the activity of AKP in the experiment was lower than the positive group, around 30 U/100 mL. From Figure 10b, the BUN content of the experimental group, around 10 mmol/L, is smaller than the positive group, which is about 12 mmol/L, in a normal range 5–15 mmol/L [48]. In each case, the PLA-PTMC results were lower than those of the positive group.

**Figure 11:**
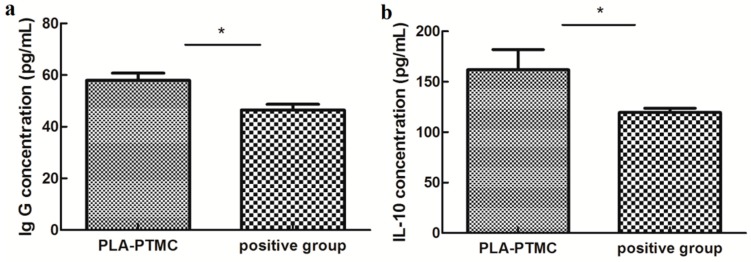
The results of ELISA testing. (**a**) is the difference of rabbit immunoglobulin G (IgG), and (**b**) is the statistical difference of rabbit interleukin 10 (IL-10). Values are the means ± S.E.M. (*n* = 5). * *p* < 0.05 compared with the control group; * *p* > 0.05 was considered not to be statistically different. These indicators were detected using kits that were obtained from CUSABIO (CUSABIO, Wuhan, China).

The immunogenic specificity of the materials was measured by ELISA as described previously [49,50]. The amounts of the cytokines TNF-α, IL-6 and IL-10 and IgG, IgM and BALP in the rabbit sera were determined using double-antibody sandwich ELISAs, and only IL-10 and IgG appeared to be statistically different between the PLA-PTMC and positive groups. IgG concentration reached to around 60 pg/mL, and IL-10 concentration was about 160 pg/mL. IL-10 is a pleiotropic cytokine that plays an important role in the development of inflammation and immune response and disease. Figure 11 shows that the expression of IL-10 and IgG in the PLA-PTMC group was higher than that in the positive control group [51]. To some degree, the experimental group was better than the positive group in terms of avoiding infection.

**Figure 12:**
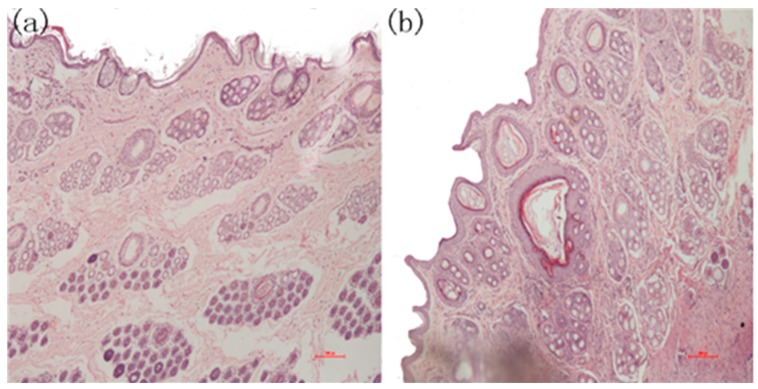
The microscopic state of the recovered wound tissue. (**a**) Skin pathological section from the PLA-PTMC group; (**b**) skin pathological section from the positive group.

During the experiments, three of the five samples in the positive group produced wound infections, which attracted our attention. After completing the initial tests, skin pathological section analyses were conducted. The tissues were excised immediately, and adhering tissues were trimmed and fixed with 4% paraformaldehyde for 24 h for the histological studies. The specimens were embedded in paraffin, and the sections were stained with hematoxylin-eosin (HE) for light microscopic observations. The sections were examined and photographed using an Olympus CX-31 Microscope (Olympus Corporation, Shinjuku, Tokyo, Japan). Three areas on each slide were chosen randomly for microscopic examination. The slides were further examined and evaluated blindly by two investigators. The images in Figure 12 indicate that there was apparent re-epithelialization that extended sufficiently to cover the wound in the PLA-PTMC group.

Figure 12 is the photomicrograph of the PLA-PTMC medical adhesive and control sites 31 days after surgery (×100). Figure 12a shows that the intact cellular structure and the fibroblastic activity of the wound in the PLA-PTMC group had both recovered 31 days after the surgery. Obviously, the positive control group had more viral factors than did the PLA-PTMC group. This was be confirmed by Majola, who suggested that PLA has no inflammation or foreign body reaction through experiments on rats [52,53], and Von Schroeder, who indicated that PLA was well tolerated with minimal inflammatory response through experiments on dogs in 1991 [54]. Thus, during the experiment, the wounds were infected more seriously in the positive control group than in the PLA-PTMC group, and inflammatory cells were more numerous than in the PLA-PTMC group. This entire process was conducted at the Yunnan University of Traditional Chinese-Medicine.

## 3. Materials and Methods

### 3.1. Synthesis of PLA-PTMC Medical Adhesive

Different ratios of poly(lactic acid) (PLA, *M*w = 280 kDa, *M*w/*M*n = 1.98) purchased from NatureWorks^®^ LLC (Minnetonka, NE, USA) and poly(trimethylene carbonate) (PTMC, *M*w = 100 kDa, *M*w/*M*n = 1.70), which was prepared in the laboratory of the Engineering Research Center of Biopolymer Functional Materials of Yunnan, Yunnan Minzu University (Kunming, China), were weighed and added to 40.0 g of ethyl acetate (>99.5%, KESHI, Chengdu, China). Dissolution required approximately 6 h in a closed environment at room temperature (approximately 25 °C).

### 3.2. Preparation of Films

PLA-PTMC medical adhesive membranes were fabricated using a solvent evaporation technique. Approximately 10.0 g of PLA-PTMC medical adhesive were weighed and dissolved in 50 mL of chloroform (>99.0%) using a batch mixer. After vigorous mixing, the film-forming solution was applied to a polytetrafluoroethylene (PTFE) plate. The solvent was allowed to evaporate at room temperature under the previous conditions to produce a PLA-PTMC medical adhesive membrane with a thickness of 0.6 mm.

The time for the pouring of the solution and for the film formation were recorded as Time 1 and Time 2, respectively. The film formation time for the PLA-PTMC medical adhesive was Time 2 minus Time 1. The composite films were then cut into 100 mm × 25.4 mm sections for investigating the film properties.

### 3.3. Water Vapor Transmission Rate

The water vapor transmission rate was determined gravimetrically using a water vapor transmission measuring cup at 35 °C and at 50% relative humidity (RH) in accordance with the ASTM E96-95 standard method [55]. Film samples were mounted over the acrylic cups and sealed with paraffin and rubber. The covered cups were placed in a constant temperature and RH-controlled chamber using the same conditions required for film equilibration. The weight loss of the measuring cup was measured as a function of time for 12 h [56]. Every sample was tested at least 8 times. The result was expressed as the average of the measurements.

### 3.4. Comfortability Degree of PLA-PTMC Medical Adhesives

According to the YY/T0471.4-2004 test methods for primary wound dressing, Part 4, comfortability, the sample was cut to a width of 25 cm, and the sample was ensured to relax freely for at least 300 s. Two parallel marks were made on the sample at a distance of 100 cm ± 0.5 mm and were recorded as *L1*, and the range of the two marks to both ends was kept equal. Finally, a Universal tensile machine (CMT4104; Power Supply 220 V; Max Force 40 KN; Accuracy 1 level; MTS SYSTEMS (CHINA) CO. LTC, Sichuan University, Chengdu, China) was used to stretch the sample at a speed of 300 mm/min, and the maximal load named (ML) was recorded when the sample was stretched by 20%. The sample was then relaxed for 1–2 s in this state, and after 5 min, the distance between the marks was recorded as *L2*. The extensibility (E) was calculated according to Formula (1), and the permanent set (PS) was calculated according to Formula (2). An average of at least five test values was obtained for each sample.

(1) $$E\;=\;\frac{ML}{2.5}$$

(2) $$\mathrm{PS}\%\;=\;\left[\left(L2-L1\right)\times \frac{100}{L1}\right]$$

### 3.5. Contact Angle

According to ISO 15989:2004 (Plastic film and sheet corona processing thin film of water contact Angle measurement) the contact angles for the film samples were measured using a Kruss Tensiometer K100 (Hamburg, Germany) at 25 °C, and the wetting characteristics of the polymer surfaces were quantified by following the Wilhelmy method. The films were doused with distilled water, dried at 25 °C and then cut into 20 × 30 mm pieces. The measurements were carried out in water at a rate of 25 µm·s^−1^. Three measurements were conducted for each sample.

### 3.6. Gel Permeation Chromatography

Gel permeation chromatography (GPC) measurements were performed on a Waters 515 system equipped with a refractive index (RI) detector using tetrahydrofuran (THF) as the solvent at a flow rate of 1.0 mL/min, and 60 µL of a 1.0 *w*/*v*% solution were injected for each analysis. Calibration was accomplished using polystyrene standards (Polysciences, Warrington, PA, USA).

### 3.7. ^1^H Nuclear Magnetic Resonance (^1^HNMR)

^1^H nuclear magnetic resonance (^1^HNMR) spectra were recorded using a Bruker AVANCE400 spectrometer (Bruker Corporation, Switzerland) operating at 400 MHz using deuterated chloroform (CDCl_3_) as the solvent. Chemical shifts (d) were obtained in ppm with respect to tetramethylsilane (TMS).

### 3.8. Differential Scanning Calorimetry

DSC analysis of PLA-PTMC medical adhesives was conducted using a DSC214 instrument (Netzsch, Selb, German) with dry nitrogen gas at a flow rate of 60 mL/min, Approximately 6 mg of each sample were placed in a small crucible with a sealed surrounding and then heated from 20–210 °C at 10 °C·min^−1^ to identify possible changes in the crystallization and in the melting transition. Subsequently, the sample was cooled to room temperature at a cooling rate of 10 °C·min^−1^ and then further heated to 220 °C at 10 °C·min^−1^.

### 3.9. Scanning Electron Microscopy

A section of the sample was sheared before performing the test. The sheared surfaces of the composite films were observed via scanning electron microscopy (SEM) under high vacuum using an SEM instrument (Nova 450, FEI Corporation, Brno, Czech Republic) in liquid nitrogen to observe the interior of the unstressed composites. To optimize the SEM examination, the sheared surfaces of samples were gold-sprayed to produce a thin conductive gold layer 5 nm thick on the exterior of the sheared surfaces.

### 3.10. Animal Experiments

The trial was approved by the laboratory animals Ethics Committee of Yunnan Minzu University (29 December 2016) and was registered on the Kunming Science and Technology Bureau (SYXK(Yunnan)K2017-0001, 16 January 2017).

#### 3.10.1. Cytotoxicity

Cell viability was determined according to ISO-10993-5 standard tests using 3-(4,5-dimethylthiazol-2-yl)-2,5-diphenyl tetrazolium bromide (MTT). Briefly, RAEC cells were seeded at 5.0 × 10^4^ cells/mL in 198 µL of appropriate culture medium containing 10% serum and 1% antibiotics in a 96-well plate and incubated for 48 h with 5% CO_2_, at 37 °C. When the cells were completely adherent, 2 µL of PLA/PTMC (D) medical adhesive at different concentrations (2.5, 5, 10, 20, 50 and 100 μg/mL) were added to the cells. At the same time, 2 μL of Dimethyl Sulphoxide (DMSO) were added to the positive control group cells. Three parallel holes were used, and the cells were incubated for 24 and 48 h. Then, 20 µL of 5 mg/mL MTT were added to the cells, and after the cells were co-incubated for 4 h, the medium was aspirated from the cells. Then, 200 µL of DMSO were added to the holes, and the plates were incubated at room temperature in the dark for 30 min and then homogenized by shaking for approximately 20 min. The formazan absorbance was measured at 570 nm using a microplate reader. A standard curve was obtained using different drug concentrations, and the growth inhibition for L6 cells was analyzed using Prism 6.0 (Nanjing Jiancheng biological technology limited company, Nanjing, China). The cytotoxicity of the PLA/PTMC (D) medical adhesive was recorded as the IC_50_.

#### 3.10.2. Test of Sterility

The same batch of PLA/PTMC (D) medical adhesive was coated evenly on the surface with the medium and then incubated in a thermostatic incubator for 36 h.

#### 3.10.3. Skin Irritability

Ten male and female rabbits were included in this experiment. The PLA/PTMC (D) medical adhesive and positive reference substance were coated on the inner ears of the rabbits and observed after 2, 4, 8, 12 and 24 h.

#### 3.10.4. Creation of the Wound Model

##### Animals and Tissue Preparation

Male and female rabbits (2.0–2.5 kg) were obtained from the Laboratory Animal Unit of Kunming Medical University (Kunming, China). All experiments performed in this study were approved by the Committee on the Use of Live Animals in Teaching and Research of Yunnan Minzu University (18 July 2016–16 November 2016). After injecting air into the ear vein of the rabbits, the wound granulation tissue and the surrounding skin on the back were excised, and the tissues were fixed immediately with 4% paraformaldehyde for 24 h.

##### Establishment of the Wound Model

Ten rabbits were randomly divided into 2 groups, with 5 rabbits in the experimental group and another 5 in the positive control group. The hair on the back of the rabbits was removed on the day before the surgery. The rabbits were anesthetized with 2 mL/kg of 10% chloral hydrate; then, a 2 cm × 2 cm square full-thickness skin wound was produced on the left side of the back, and a full-thickness skin scratch of a length of 2 cm was created on the right side of the back. The surgical site and scratches were treated with the PTMC medical adhesives (0.5 mm-thick membranes) and the positive control drug, respectively. After surgery, the food intake, wound granulation tissue growth and inflammation of the rabbits were observed, and the temperature was measured.

Wound healing rate was one of the direct indicators of wound healing. In our work, the wound healing rate was evaluated as in the work of Nagelschmidt’s and Pulok K. Mukherjee’s group [56,57]. The changes in healing of the wound, namely the measurement of wound area on graph paper, was expressed as units of mm^2^. The healing rate of the wounds was expressed as Formula (3).

(3) $$\mathrm{Healing}\;\mathrm{rate}\;\mathrm{of}\;\mathrm{wound}\;=\;\frac{\;\mathrm{original}\;\mathrm{area}-\mathrm{unhealed}\;\mathrm{wound}\;\mathrm{area}}{\mathrm{original}\;\mathrm{area}\;}$$

##### Weight and Temperature Transform

On the day of operation, the weights of the rabbits that were used in the wound experiments were obtained and recorded. The weights were also recorded on the 31st day. In addition, rectal temperatures were recorded once per day after the operation and, after four days, once per three days until the wound recovered completely.

##### Hepatic and Renal Function

On the 31st day after the wounds were created, 15 mL of blood gained was collected from the heart and prepared for next further study. Serum was prepared by centrifugation for 5 min at 37 °C at 4500 rpm. The hepatic indexes, including AST/GOT, ALT/GPT, AKP, BUN and Cr, were determined using special kits (Nanjing Jiancheng biological technology limited company, Nanjing, China), according to the manufacturer’s instructions.

##### ELISA Assay

IgM, IgG, BALP, IL-6, IL-10 and TNFα were determined in the rabbit serum using double-antibody sandwich ELISAs (CUSABIO), according to the manufacturer’s instructions.

##### Skin Pathology Analyses

Skin pathological section analyses were performed after the other tests were completed. The tissues were excised, and adhering tissues were trimmed and then fixed with 4% paraformaldehyde for 24 h for histological studies. The specimens were embedded in paraffin and sectioned, and the sections were stained with hematoxylin-eosin (HE) for light microscopic observations. The sections were examined and photographed using an Olympus CX-31 Microscope (Olympus Corporation, Shinjuku, Tokyo, Japan).

### 3.11. Statistical Analysis

All data were expressed as the means ± S.E.M. Statistical analyses were performed using Prism 6.0. Comparisons between two groups were performed using unpaired tests. Comparisons among three or more groups were performed using a one-way ANOVA. *p* < 0.05 was considered statistically significant, and *p* > 0.05 was considered to have no statistically-significant difference.

## 4. Conclusions

In this study, an adhesive that reduces infections effectively because of its breathability was prepared from PLA modified with PTMC via blending at room temperature. Through a series of characterizations of the performance of different ratios of PLA and PTMC, an optimal ratio was determined, and the effectiveness of the best ratio of PLA and PTMC was demonstrated through animal experiments. The results suggest that a ratio of PLA to PTMC of 7:3 produces a material with suitable gas permeability that can reduce the incidence of wound infections. Furthermore, it produces no harm to the liver and kidneys. It also promotes the proliferation of L6 cells, fibroblasts and epidermal stem cells within the skin wound. Therefore, a blend of PLA and PTMC at a ratio of 7:3 has promise as a medical adhesive.

## Figures and Tables

**Table 1:** Molecular characteristics of poly(lactic acid) (PLA) and PLA/PTMC (poly(lactic acid)/poly(trimethylene carbonate)) blends with different ratios.

Groups	PLA/PTMC ^a^	Solvent	Temperature (°C)/Time (h)	*M*n ^b^	PDI ^b^
A	10:0	ethyl acetate	25/6	98,972	2.052
B	9:1	ethyl acetate	25/6	93,193	1.957
C	8:2	ethyl acetate	25/6	89,888	1.871
D	7:3	ethyl acetate	25/6	77,253	1.846

^a^ Calculated from ^1^HNMR data using CDCl_3_ as the solvent; ^b^ determined from GPC data. PDI: polydispersity index.

**Table 2:** Contact angle measurement.

Group	Contact Angle (Degree)
PLA	72.45
PLA-PTMC 9:1	74.96
PLA-PTMC 8:2	80.75
PLA-PTMC 7:3	87.38
